# Kinetics of alpha-synuclein depletion in three brain regions following conditional pan-neuronal inactivation of the encoding gene (*Snca*) by tamoxifen-induced Cre-recombination in adult mice

**DOI:** 10.1007/s11248-021-00286-3

**Published:** 2021-09-29

**Authors:** Kirill D. Chaprov, Ekaterina A. Lysikova, Ekaterina V. Teterina, Vladimir L. Buchman

**Affiliations:** 1 Institute of Physiologically Active Compounds Russian Academy of Sciences (IPAC RAS), 1 Severniy proezd, Chernogolovka, Moscow Region Russian Federation 142432; 2 School of Biosciences, Cardiff University, Museum Avenue, Cardiff, CF10 3AX UK

**Keywords:** Alpha-synuclein, Conditional gene knockout, Tamoxifen-induced Cre-recombination, Striatum, Midbrain, Cerebral cortex

## Abstract

Conditional pan-neuronal inactivation of the *Snca* gene in 2-month old male and female mice causes dramatic decrease in the level of the encoded protein, alpha-synuclein, in three studied brain regions, namely cerebral cortex, midbrain and striatum, 12 weeks after the last injection of tamoxifen. Kinetics of alpha-synuclein depletion is different in these brain regions with a longer lag period in the cerebral cortex where this protein is normally most abundant. Our results suggest that efficient post-developmental pan-neuronal knockout of alpha-synuclein in adult, i.e. 5- to 6-month old, animals, could be achieved by tamoxifen treatment of 2-month old mice carrying loxP-flanked *Snca* gene and expressing inducible Cre-ERT2 recombinase under control of the promoter of neuron-specific enolase (NSE) gene.

**Supplementary Information:**

The online version contains supplementary material available at 10.1007/s11248-021-00286-3.

## Introduction

A pivotal role of alpha-synuclein in molecular pathogenesis of several neurodegenerative diseases including one of the most common, Parkinson’s disease, is well established (Bras et al. 2020). Genetically altered mouse lines overexpressing native alpha-synuclein or expressing its pathogenic variants either pan-neuronally or in specific neuronal and glial cell populations were important tools for revealing principal mechanisms of alpha-synuclein malfunction in pathology (Buchman and Ninkina 2008; Dehay and Fernagut 2016; Koprich et al. 2017). The first mouse line with targeted inactivation of the alpha-synuclein-encoding *Snca* gene has been described two decades ago (Abeliovich et al. 2000), and since that time several more mouse lines with constituent knockout of this gene have been produced and used in multiple studies that significantly improved our understanding of alpha-synuclein biological functions and a potential role of its loss-of-function in pathological processes affecting synaptic transmission (for recent reviews see Burre et al. 2018; Longhena et al. 2019; Sulzer and Edwards 2019). However, interpretation of data obtained in studies of mice with constituent inactivation of the *Snca* gene can be ambiguous. For example, studies of functional consequences of alpha-synuclein depletion in the mature nervous system might be affected by the developmental compensation of alpha-synuclein loss by other members of the synuclein family and, potentially, other proteins/mechanisms. Moreover, changes observed in a studied neuronal population or network could be caused not by alpha-synuclein depletion in this population/network but be an indirect consequence of its depletion in another region of the nervous system or in another body system where alpha-synuclein is expressed, e.g. certain types of blood cells.

An ability to inactivate the *Snca* gene in a spatially and temporally regulated manner could help to clarify most uncertainties about normal function of alpha-synuclein in specific types of cells. In addition, conditional inactivation of the *Snca* gene in the nervous system of ageing mice creates a model of the depletion of functional alpha-synuclein in neurons of patients with alpha-synucleinopathy due sequestration of this protein in pathological inclusions. Furthermore, conditional inactivation of the gene could create much-needed controls for many experiments studying propagation of alpha-synuclein pathology throughout the nervous system and testing anti-alpha-synuclein aggregation drugs. Recently, we have produced a mouse line with the first coding exon of the *Snca* gene flanked by loxP sites and confirmed that Cre-recombination leads to complete inactivation of the gene—when such inactivation was triggered in the germ line of “floxed” mice it created a novel “low footprint” constituent knockout line (Ninkina et al. 2015; Goloborshcheva et al. 2020). Further studies demonstrated that 4 months after conditional pan-neuronal inactivation of the *Snca* gene by tamoxifen-induced Cre-ERT2-driven recombination in adult (6-month old) or ageing (12-month old) mice alpha-synuclein becomes virtually undetectable in the striatum (Ninkina et al. 2020), suggesting that these mice could be successfully used for certain ageing studies.

However, 4- to 6-month old, aka ‘adult’, mice are more commonly used in various behavioural, physiological, morphological and biochemical studies. If conditional inactivation of a gene in a particular type of cells is considered for these type of studies, and potential developmental effects of such inactivation need to be avoided, it is important to know (i) how long it takes for the encoded protein to be depleted from the studied cell population, i.e., when a knockout state of these cells is achieved after triggering gene inactivation, and (ii) at what age gene inactivation could be triggered to achieve this knockout stage in ‘adult’ mice. Therefore, here we investigated if triggering of *Snca* gene inactivation at the age of 2 months, when the development of mouse nervous system is essentially completed, would leave sufficient time for efficient depletion of alpha-synuclein from neurons of different brain regions before animals reach the age of 4–6 months.

## Materials and methods

### Animals

Three original mouse lines were used in the process of production of experimental and control animals: a conditional knockout *Snca*^flox/flox^ line (Ninkina et al. 2015), deposited to The Jackson Laboratory as C57BL/6-*Snca*^*tm1.1Vlb*^/J, JAX Stock # 025636; a “low footprint” constituent knockout *Snca*^Δflox/Δflox^ line (Ninkina et al. 2015), deposited to The Jackson Laboratory as B6(Cg)-*Snca*^*tm1.2Vlb*^/J, JAX Stock # 028559; a transgenic line carrying a cassette for expression of Cre-ERT2 recombinase under control of a neurospecific NSE promoter (obtained from Jean C. Manson, University of Edinburgh). All three lines were previously established on and maintained on a pure C57Bl6J (Charles River) genetic background in the UK laboratory. Cohorts of experimental animal were produced form these core lines in the Russian laboratory where animals were housed in a specific pathogen free facility at temperature 21 ± 2 °C and 40–60% humidity with 12/12 h light/dark cycle. Throughout the study animals had ad libitum access to food and water.

### *Snca* gene inactivation

All mice born from the final cross required for production of experimental and control animals should be of the same desired genotype. However, we still genotyped all animals participated in the study using genotyping protocols for detection of the *Snca* gene modifications and for the presence of the NSE/Cre-ERT2 transgenic cassette as described in our previous publications (Ninkina et al. 2015, 2020; Roman et al. 2017). Male and female littermates were randomly selected for experimental and control group. Experimental animals received 5 daily i.p. injections of tamoxifen (0.5 mmol/kg dissolved in corn oil) and control animals received vehicle injections. At selected time points mice were euthanised by cervical dislocation and brain regions were dissected.

### Detection of alpha-synuclein in mouse brain samples

Mice were euthanised by a Schedule 1 method, brains removed, and brain regions dissected as described in numerous publications, for example in Spijker (2011), for a typical image of brain prepared for dissection with regions of interest outlined see Online Resource 1. Preparation of total protein lysates from dissected brain regions and Western blot analysis were carried out as described previously (Connor-Robson et al. 2016; Ninkina et al. 2020). Highly specific mouse monoclonal antibodies 4D6 (Abcam), TH-2 and AC-74 (both Sigma) were used for detection of alpha-synuclein, tyrosine hydroxylase and beta-actin protein bands, respectively. Statistical significance for relative abundance of alpha-synuclein in studied samples was calculated only for differences between control and 12 weeks groups for which more samples were analysed (n = 6–9) than for other age groups (n = 3).

## Results and discussion

Cohorts of experimental and control animals were produced by cross-breeding two parental mouse lines. The line of mice homozygous for the *Snca* gene containing a floxed first coding exon (*Snca*^flox/flox^) has been previously produced in our laboratory (Ninkina et al. 2015). The second line was similar to the line used in our previous study (Ninkina et al. 2020): homozygous for both constituently inactivated *Snca* gene and a transgenic cassette for expression of the tamoxifen-inducible Cre-ERT2 recombinase under control of a pan-neuronal NSE promoter. However, for production of the new parental line for this study (L5 line: *Snca*^Δflox/Δflox^ + NSE/Cre-ERT2) a “low footprint” constituent knockout parental line B6(Cg)-*Snca*^*tm1.2Vlb*^/J (Ninkina et al. 2015) was used instead of the knockout line originally described by Abeliovich et al. (2000). This prevents any possible effects that might develop as the result of substantially increased level of expression of the *Mmrn1* (Goloborshcheva et al. 2020) and potentially, other genes located in close proximity to the *Snca* gene due to the presence of neo expression cassette in the modified *Snca* genomic locus of mice produced by Abeliovich and colleagues. The new parental line used here is yet to be deposited to The Jackson Laboratory but currently is available from authors by request.

**Fig. 1:**
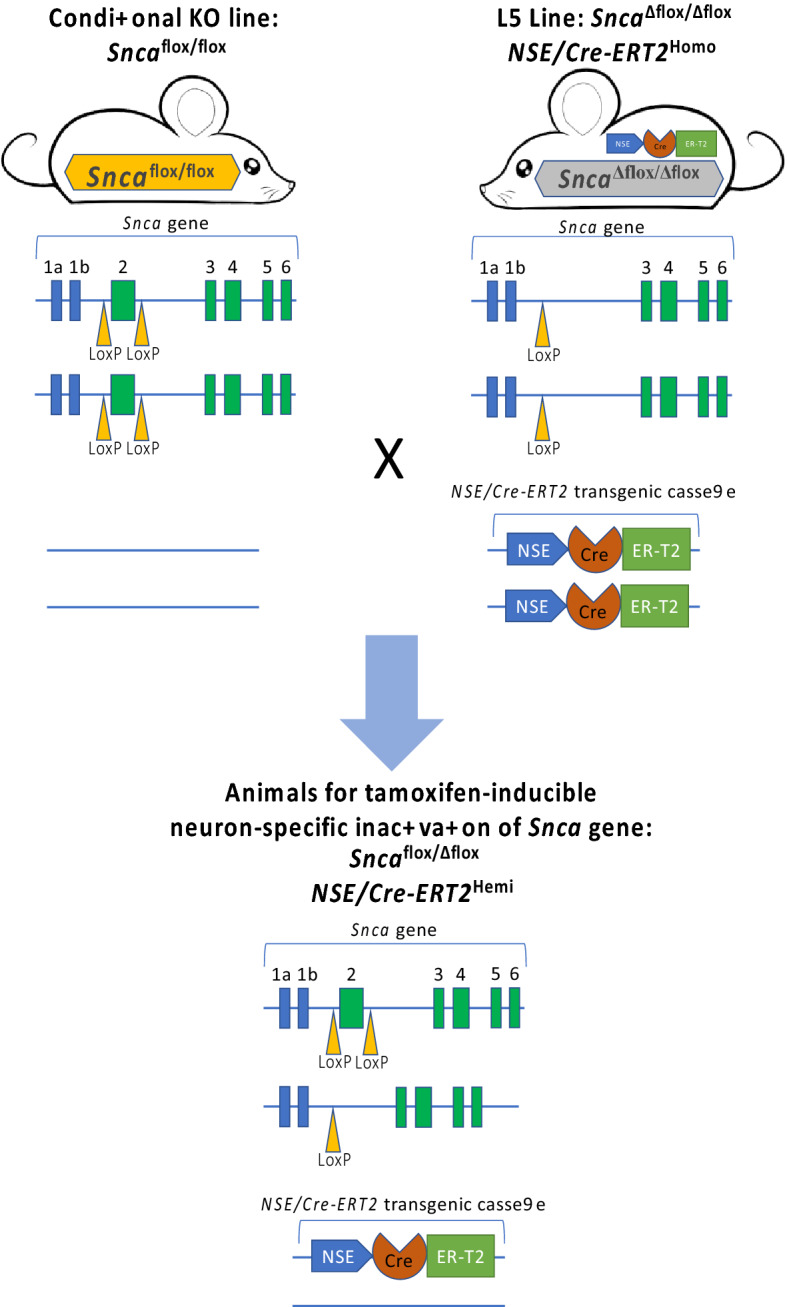
Scheme demonstrating production of animals for analysis of alpha-synuclein depletion following tamoxifen-inducible inactivation of the encoding gene. All littermates from the crossbreeding of two parental lines share the same genotype that allows complete cessation of alpha-synuclein production in neurons following activation of loxP/Cre recombination

As illustrated in Fig. 1, all animals obtained from crossing these two parental lines (thereafter “floxed mice”) contained a floxed first coding exon in one copy of the *Snca* gene with another copy of the gene constituently inactivated, and also carried a transgenic cassette for expression of the tamoxifen-inducible Cre-ERT2 recombinase under control of a pan-neuronal NSE promoter. Thus, inactivation of the floxed copy of the *Snca* gene by loxP/Cre recombination following tamoxifen treatment creates a knockout genotype in neurons.

**Fig. 2:**
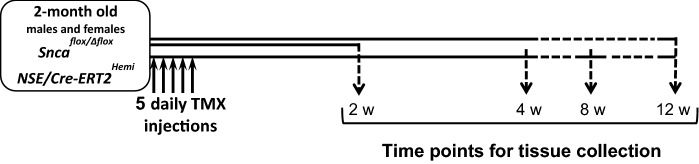
Workflow of the experimental setup for animal treatments and collection of brain tissues for further analysis

**Fig. 3:**
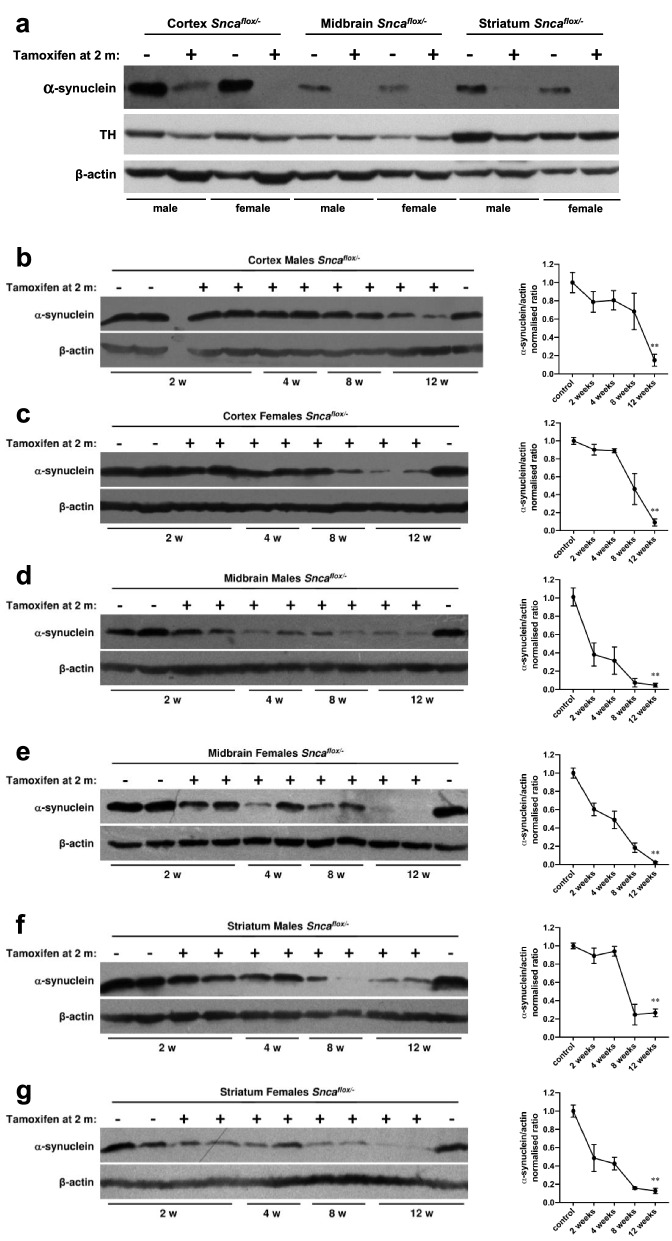
Depletion of alpha-synuclein from mouse brain regions following conditional inactivation of the encoding gene in neurons of 2-month old animals. Levels of alpha-synuclein in three brain regions were analysed by Western blotting at different time point after tamoxifen treatment of 2-month old male and female mice carrying a floxed *Snca* gene and a transgenic cassette driving expression of Cre-ERT2 under control of neurospecific NSE promoter. Littermate animals of the same sex and genotypes that did not receive tamoxifen we used as control groups. **a** A representative Western blot shows dramatically reduced levels of alpha-synuclein but not of tyrosine hydroxylase in three brain regions 12 weeks after tamoxifen treatment. **b**–**g** Representative Western blots and corresponding graphs show kinetics of alpha synuclein depletion separately in each of three brain regions of male and female animals. Statistical significance shown (**p < 0.01, Mann–Whitney U-test) was calculated only for differences between control and 12 weeks groups for which more samples were analysed (n = 6–9) than for other age groups (n = 3), which was not sufficient for proper statistical analysis

At the age of 2 months male and female “floxed mice” were treated with tamoxifen or vehicle as described in the “Materials and methods” section and illustrated in Fig. 2. The abundance of alpha-synuclein in the cerebral cortex, midbrain and striatum of these mice was first assessed 12 weeks after the last tamoxifen or vesicle injection by Western blot analysis of total protein lysates. At this time point substantial and highly statistically significant depletion of alpha-synuclein was observed in all three brain regions of male and female animals (Fig. 3 and Online Resource 2). In some samples, alpha-synuclein was virtually undetectable even on overexposed blots whereas in other samples, a prominent band was observed. One explanation for this variability is that in our experimental system only neurons become depleted of alpha-synuclein whereas this protein is also present, although at lower levels, in certain other types of cells in the nervous system and the amount of nonneuronal alpha-synuclein might be different between regions and individual samples.

To assess the kinetics of alpha-synuclein depletion the level of this protein in each of three studied brain regions was evaluated at three additional time points: 2, 4 and 8 weeks after the last tamoxifen injection. We found that this kinetics was generally similar for male and female animals in each of the studied brain regions but slightly different between these regions (Fig. 3b–g).

Results of our study clearly demonstrate that although the kinetics of alpha-synuclein depletion following tamoxifen-induced inactivation of *Snca* gene in neurons may vary slightly between individual adult animals, only a small fraction of alpha-synuclein remains in three studied brain regions of all of these animals 12 weeks after triggering Cre-recombination at the age of 2 months. Therefore, we recommend injecting *Snca* gene floxed/Cre-ERT2-expressing mice with tamoxifen at least 3 months before commencing any experiments that require conditional depletion of alpha-synuclein in neurons of adult mice.

## Supplementary Information

Below is the link to the electronic supplementary material.

**Online Resource 1** Illustration of brain dissection used for obtaining samples of different brain regions for assessing alpha-synuclein abundance in these regions. Dashed lines show approximate positions of cuts used to dissect brain regions. The colour of the line corresponds to the colour of highlighted name of the region.

**Online Resource 2** Depletion of alpha-synuclein in the cerebral cortex, midbrain and striatum 12 weeks after tamoxifen-induced inactivation of the Snca gene gene in 2-month old “floxed mice”. Images of representative Western blots used for analysis of the abundance of α-synuclein in the total protein samples of the cerebral cortex, midbrain and striatum. Levels of alpha-synuclein were analysed 12 weeks after tamoxifen treatment of 2-month old male and female mice carrying a floxed Snca gene and a transgenic cassette driving expression of Cre-ERT2 under control of neurospecific NSE promoter. Littermate animals of the same sex and genotypes that did not receive tamoxifen we used as control groups.

## Data Availability

All data generated or analysed during this study are included in this published article and its supplementary information file.

## Abbreviations

NSE: Neuron-specific enolase
Cre-ERT2: Tamoxifen inducible Cre-estrogen receptor fusion protein
