# Supplementary figures and images for: Kinetics of alpha-synuclein depletion in three brain regions following conditional pan-neuronal inactivation of the encoding gene (Snca) by tamoxifen-induced Cre-recombination in adult mice
Source: Transgenic Res. 2021 Sep 29;30(6):867–73. doi: 10.1007/s11248-021-00286-3 (PMC8580898; doi:10.1007/s11248-021-00286-3)

Striatum, Midbrain and Cortex dissections


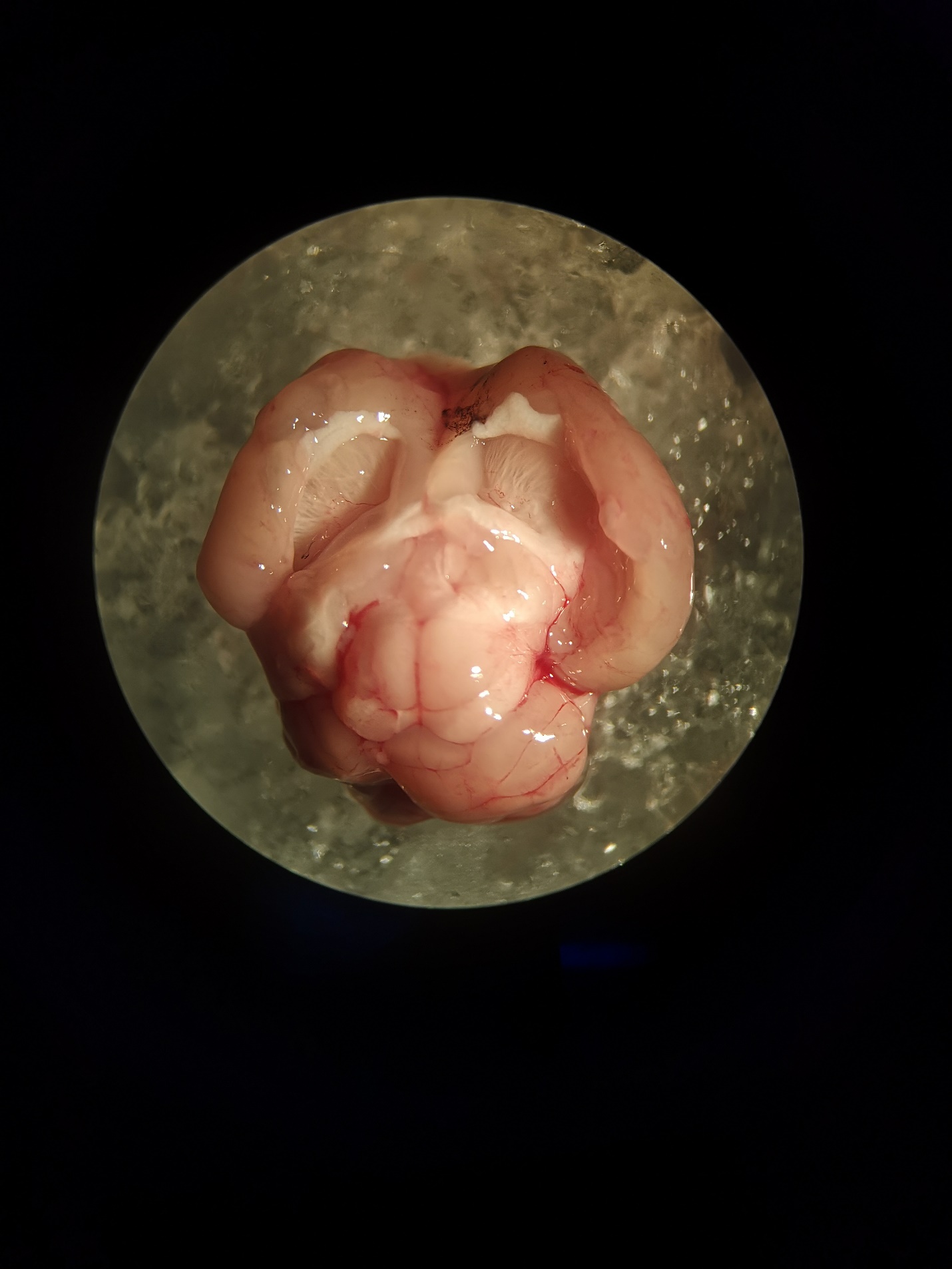

Supplement: Supplementary file 1 — Online Resource 1 Illustration of brain dissection used for obtaining samples of different brain regions for assessing alpha-synuclein abundance in these regions. Dashed lines show approximate positions of cuts used to dissect brain regions. The colour of the line corresponds to the colour of highlighted name of the region. [file 11248_2021_286_MOESM1_ESM.docx]
