# Supplementary material for: Kinetics of alpha-synuclein depletion in three brain regions following conditional pan-neuronal inactivation of the encoding gene (Snca) by tamoxifen-induced Cre-recombination in adult mice
Source: Transgenic Res. 2021 Sep 29;30(6):867–73. doi: 10.1007/s11248-021-00286-3 (PMC8580898; doi:10.1007/s11248-021-00286-3)
Supplement: Supplementary file 2 [file 11248_2021_286_MOESM2_ESM.docx]

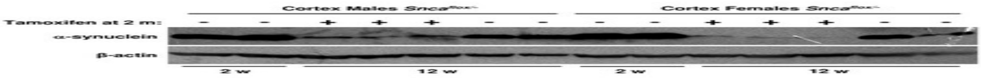
Cortex Males Snca^flox/-
Cortex Females Snca^flox/-
Tamoxifen at 2 m: - - + + + - - - - + + + - -
α-synuclein
β-actin
2 w
12 w
2 w
12 w

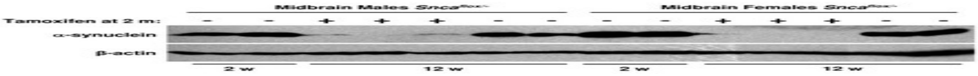
Midbrain Males Snca^flox/-
Midbrain Females Snca^flox/-
Tamoxifen at 2 m: - - + + + - - - - + + + - -
α-synuclein
β-actin
2 w
12 w
2 w
12 w

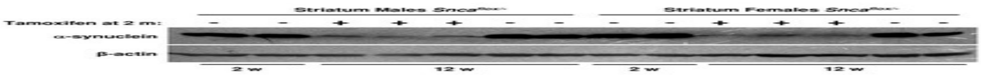
Striatum Males Snca^flox/-
Striatum Females Snca^flox/-
Tamoxifen at 2 m: - - + + + - - - - + + + - -
α-synuclein
β-actin
2 w
12 w
2 w
12 w
